# Automated Subfield Volumetric Analysis of Hippocampus in Patients with Drug-Naïve Nondementia Parkinson's Disease

**DOI:** 10.1155/2019/8254263

**Published:** 2019-02-03

**Authors:** Jin-Woo Park, Chan-Nyoung Lee, Youngbo Sim, Hyung-Kyu Ham, Woo-Suk Tae, Byung-Jo Kim

**Affiliations:** ^1^Department of Clinical Pharmacology and Toxicology, Korea University College of Medicine, Korea University Anam Hospital, Seoul, Republic of Korea; ^2^Department of Neurology, Korea University College of Medicine, Korea University Anam Hospital, Seoul, Republic of Korea; ^3^Brain Convergence Research Center, Korea University, Seoul, Republic of Korea; ^4^Saint John's Preparatory School, Collegeville, MN, USA; ^5^Neuroscience Research Institute, Kangwon National University College of Medicine, Kangwon, Republic of Korea

## Abstract

Several studies used automated segmentation of hippocampal subfield (ASHS) for detailed measurements of anatomic subregions of the hippocampus, especially in the field of neurodegenerative disorders. In this study, we investigated the hippocampal subfield volume of patients with early-stage nondementia PD compared with normal healthy subjects using the ASHS method. A total of 32 subjects were enrolled in this study (sixteen patients with drug naive nondementia PD and sixteen healthy controls). All subjects were scanned with a 1.5 tesla MRI. The volumes of the seven subfields were calculated separately, and then, the whole hippocampal volume was calculated by the summing of CA1, CA2-3, CA4-DG, subiculum, presubiculum, and fimbria, excluding the hippocampal fissure. There were significant diagnosis-by-hemisphere interactive effects on the total hippocampal volume (*F* = 5.197; *p*=0.031) and the subfield volume of CA2-3 (*F* = 7.586; *p*=0.010) and CA4-DG (*F* = 7.403; *p*=0.011). The volumes of CA2-3 (*F* = 19.911; *p* < 0.001), CA4-DG (*F* = 20.273; *p* < 0.001), and total hippocampus (*F* = 10.573; *p*=0.005) in the left hemisphere were reduced compared to the right hemisphere. We suggest that the hippocampal volume asymmetry, especially in CA4-DG and CA2-3, could be observed in drug-naïve PD patients even in the early stage of the disease.

## 1. Introduction

Parkinson's disease (PD) is one of the common neurodegenerative disorders, presenting with various clinical manifestations and heterogenous disease progression [1, 2].

While F-18 FP-CIT PET imaging has been widely accepted as a standard tool for pathophysiologic evaluation of patients with PD, conventional MRI is less often used in clinical practice; especially in the early diagnosis of PD, its specificity and sensitivity are not satisfactory [3]. However, recent studies revealed that analysis of MRI using voxel-based morphometry (VBM), functional MRI (fMRI), and diffusion tensor imaging (DTI) provides potentially useful information regarding disease progression [3, 4]. More recently, several studies used automated segmentation of hippocampal subfield (ASHS) for detailed measurements of anatomic subregions of the hippocampus, especially in the field of neurodegenerative disorders including PD [5–7]. These studies suggested that the changes in the hippocampal subfields could be associated with cognitive functions and could be used as informative diagnostic biomarkers for PD dementia (PDD) and PD with mild cognitive impairment (PD-MCI) [6–8].

Although there have been several reports suggesting hippocampal atrophy could be observed in the early stage of PD [7, 9], there were no studies evaluating hippocampal volume loss that focused on drug-naïve PD without cognitive dysfunction using the ASHS analysis method. As mild cognitive impairment (MCI) presents as one of the earliest premotor symptoms of PD, we hypothesized that there could be hippocampal asymmetry in early PD patients compared to normal healthy controls and the manifestation might be different from well-known CA1-2 atrophy in early Alzheimer's disease (AD). Therefore, in this study, we investigated the hippocampal subfield volume of patients with drug-naïve early-stage nondementia PD compared with normal healthy subjects using the ASHS method.

## 2. Methods

### 2.1. Subjects

This study was performed prospectively. Sixteen patients with drug-naïve nondementia PD according to the United Kingdom Parkinson's Disease Society brain bank diagnostic criteria were recruited consecutively in their first visit to the neurology clinic. Patients with Hoen and Yahr stage (H&Y stage) I, II, or III were included. Exclusion criteria were the following: a history of head trauma, stroke, exposure to antidopaminergic drugs, central nervous system infection, abnormal thyroid function, a structural lesion or hydrocephalus on brain MR imaging, and red flag signs suggestive of PD plus syndrome or dementia (MMSE score of <24). Sixteen sex-, handedness-, and age-matched healthy volunteers were used as control subjects. The clinical assessment of patients included the MMSE, UPDRS part III, and H&Y staging. The present study was approved by the Ethics Committee of the university-affiliated hospital. All patients and volunteers gave informed consent to participate in the study, and the study protocol was approved by the institutional review board (IRB) of the hospital. The demographics of the participating patients and control subjects are described in Table 1.

### 2.2. MRI Acquisition

All patients with PD and normal controls were scanned with a 1.5 tesla MRI (Gyroscan ACS-NT; Philips Medical Systems, Best, the Netherlands). To maintain consistent positioning of individuals' heads, the pilot images of coronal, sagittal, and axial T1 MR images with fast-field echo sequence were acquired using the following MRI scanning variables: slice thickness = 10 mm with a gap of 10 mm, repetition time (TR/echo time (TE)) = 15/5.2 msec, in-plane matrix = 256 × 256, field of view (FOV) = 25 × 25 cm, 4 slices in each orientation, and flip angle = 20°.

Coronal 3D T1-weighted turbo field echo MRI data were acquired with the following protocols: slice thickness = 1.3 mm without gap, number of slices = 160, scan time = 10 min 13 sec, TR/TE = 10/4.3 msec, number of excitations = 1, image matrix = 256 × 256, FOV = 22 × 22 cm^2^, and flip angle = 8°. To correct the head tilts in the MRI bore, coronal 3D T1 MRI was performed perpendicular to the long axis from the anterior commissure to the posterior commissure in the midsagittal plane of the interhemispheric commissure before MRI scans. The voxel size of the 3D MRI was 0.86 × 0.86 × 1.30 mm^3^ (*x* × *y* × *z*, respectively).

### 2.3. Image Processing

**Figure 1:**
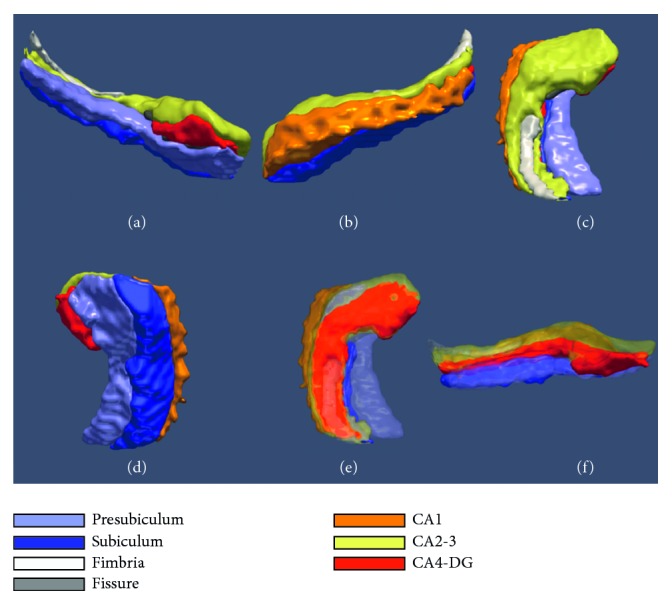
Hippocampal subfields in various views from a magnetic resonance image from a patient with PD. (a) Medical view. (b) Lateral view. (c) Superior view. (d) Inferior view. (e-f) Transparent view.

To regress out the effect of habitual brain subvolumes, the total intracranial cavity volumes (TICV) of all subjects were manually quantified using the Cavalieri method [10], and the reproducibility of ICV (Cronbach *α* = 0.996) has been previously reported [11]. Using the automated hippocampal subfield segmentation method of van Leemput et al. [12] included in the FreeSurfer 5.3 (Massachusetts General Hospital, Boston, U.S., http://surfer.nmr.mgh.harvard.edu), each hippocampus image from the 3D T1 MRI was segmented into the following subfields: the cornu ammonis CA1, CA2-3, CA4, and dentate gyrus (DG), subiculum, presubiculum, fimbria, and hippocampal fissure, which is filled with cerebrospinal fluid. The volumes of the seven subfields were calculated separately, and then, the whole hippocampal volume was calculated by the summing of CA1, CA2-3, CA4-DG, subiculum, presubiculum, and fimbria, excluding the hippocampal fissure [13] (Figure 1). The technical procedures and details of this automated segmentation of hippocampal subfields have been previously described [12].

### 2.4. Statistical Analyses

We compared the automatically calculated total hippocampal volume and subfield volumes for the two diagnosis groups (PD group vs. healthy control group) and the hemispheres (left vs. right) and investigated whether there was a diagnosis-by-hemisphere interaction. Repeated-measures analysis of covariance (RM-ANCOVA) was used in the analysis with the total hippocampal volume and subfield volumes as the dependent variables, the diagnosis group and hemisphere as the independent variables, and age, sex, and TICV as the covariates. To correct for multiple comparisons, the false discovery rate (FDR) introduced by Benjamini and Hochberg [14] was applied in the comparisons of total and subfield volumes in patients with PD vs. healthy controls (8 comparisons, *q* < 0.05). In the post hoc analysis, we compared any of the total or subfield volumes that had a significant diagnosis-by-hemisphere interaction using a repeated-measures analysis of variance (RM-ANOVA). The between-group differences in the demographic and clinical characteristics of the patients with PD and healthy controls were analyzed using *t*-tests for age and the score of MMSE, and the distribution of sex was analyzed with a chi-square test. Statistical analyses were performed using SPSS version 22.0 (SPSS Inc., Chicago, IL, USA).

## 3. Results

### 3.1. Demographic and Clinical Characteristics

All participants were right handed. The age, sex distribution, MMSE scores, disease duration, UPDRS, H&Y stage, and TICV of 16 patients in PD group and 16 healthy controls are shown in Table 1. There were no significant differences between the groups in the mean age (*p*=0.606), sex distribution (*p*=1.000), the scores of MMSE (*p*=0.073), or TICV (*p*=0.678).

### 3.2. Comparison of Total and Subfield Hippocampal Volume

In the main analysis, we found that the diagnosis group and hemisphere were not related to the total hippocampal volume and subfield volumes, but there were significant diagnosis-by-hemisphere interactive effects on the total hippocampal volume (*F* = 5.197; *p*=0.031) and the subfield volume of CA2-3 (*F* = 7.586; *p*=0.010) and CA4-DG (*F* = 7.403; *p*=0.011), as shown in Table 2. The diagnosis-by-hemisphere interaction in the volume of CA4-DG remained significant after the FDR correction.

We performed a post hoc analysis of the volumes with significant diagnosis-by-hemisphere interaction using RM-ANOVA. In the PD group, the volumes of CA2-3 (*F* = 19.911; *p* < 0.001), CA4-DG (*F* = 20.273; *p* < 0.001), and total hippocampus (*F* = 10.573; *p*=0.005) in the left hemisphere were reduced compared to the right hemisphere (Table 3). There were no significant differences in the volumes of CA2-3, CA4-DG, and total hippocampus between the left and right hemispheres in the healthy control group (all *p* > 0.1).

## 4. Discussion

Our study highlighted that hippocampal asymmetry, especially in CA4-DG and CA2-3, could be observed in patients with drug-naïve PD by using the ASHS method. The CA4-DG area appeared to be the most affected after FDR correction.

Although possible hippocampal atrophy in patients with nondementia PD has been discussed over many years, there was controversy over the issue [6, 7, 9, 15–17]. Our finding was consistent with the previous pathologic study, in which Braak et al. suggested that atrophy of CA2 and hippocampal Ammon's horn are among the characteristics of PD pathology [18]. Moreover, the results were distinct from previous findings in the study of early Alzheimer's disease (AD), in which hippocampal atrophy of CA1-2 was the most predominant feature [19–21]. For example, Kerchner et al. argued that CA1 stratum radiatum and stratum lacunosum moleculare (SRLM) could occur in the early stage of AD, and this area is related to memory loss in patients with AD. This finding was likely correlated with Braak's finding in 1991, which suggested that neurofibrillary tangles and neuropil threads were observed in CA1 in AD pathology [22].

We were curious about our result indicating that the CA4-DG area showed the most asymmetry related to drug-naïve PD, as this area was not known to be related to early-stage PD. However, Pereira et al. mentioned that they observed significant volume atrophy in the CA2-3 and CA4-DG subfields of patients with PD, similar to our study. They also commented that these findings are reasonable, considering the pathologic findings of previous studies and currently known functional distinctions of the hippocampal subfields, in particular CA2-3 and DG engagement in learning and creating new memories [7]. More recently, Tanner et al. also reported hippocampal morphometric analysis results including possible primary atrophy of CA3-4 and dentate gyrus in nondemented PD patients [9], and they argued that this could be a possible implication for white matter connectivity considering the previous report showing entorhinal cortex atrophy in PD patients with normal cognition [23]. Additionally, in a transgenic mouse model of early PD demonstrated that alpha-synuclein-positive cells were primarily located in the DG, CA3, and CA2 regions [24]. These results are consistent and support our study, and they suggest the possible presence of DG and CA4 pathology in early PD patients. Further studies of detailed hippocampal subfield evaluations in early PD are needed to understand the serial degeneration of the regions already indicated by pathology studies.

We also observed more significant volume asymmetry of CA4-DG and CA2-3 in the dominant hemisphere of the PD patients (the left hemisphere). Although previous study suggested that hippocampal asymmetry could be observed in normal healthy subjects (right > left) [25, 26], Woolard and Heckers suggested that the volume reduction was primarily observed in anterior hippocampus, mostly dominated by CA1 and subiculum [25]. The volume reduction mainly observed in CA4-DG in our study may be a suggestive of finding other than the usual hippocampal appearance. This laterality trend of dominant hemisphere is also consistent with the results of an earlier study suggesting dominant hemisphere-related vulnerability of the brain, which could strengthen our result [27, 28]. However, we could not match these findings with clinical manifestations due to the scarcity of data.

This study has several limitations. The study has a relatively small sample size and a lack of detailed cognitive function data other than the MMSE. According to the small sample size, the detailed further analysis was limited including comparison depending on nonmotor symptoms preceding PD, PD onset, and relationship between MMSE score and hippocampal subregional volumes. However, this sample size is sufficient to suggest and strengthen our basic hypothesis, as did similar studies [6, 7]. The mean MMSE of the patients with drug-naive PD was 27.40 ± 1.24. This score is accepted as an indicator of normal cognitive function, considering the patients' age (68.25 ± 7.22 years), and the score was not statistically different from that of healthy controls. Moreover, no patients or caregivers reported any problems related to cognitive function. Lastly, although our data were collected from 1.5-T MRI, the hippocampal borders could be distinguished sufficiently for the detailed analysis [29].

In conclusion, the hippocampal asymmetry of CA4-DG and CA2-3 can be observed in patients with drug-naïve nondementia PD by using the ASHS method. As there is a scarcity of studies of the area of the hippocampus and surrounding regions, we believe that future similar research would broaden our knowledge in the pathophysiology of PD and will advance imaging technologies. A serial follow-up study and pathology-based study should be performed to confirm our study results, and the conversion rate to PD-MCI or PDD in these types of patients should be examined carefully in those studies.

## Figures and Tables

**Table 1:** Demographic and clinical characteristics of patients with drug-naïve nondementia Parkinson's disease and healthy controls.

	HC (*N*=16)	PD (*N*=16)	*p* value
Age (years)	69.5 ± 6.3	68.25 ± 7.22	0.606
Sex (%, female)	13 (81.3%)	13 (81.3%)	1.000
MMSE	27.90 ± 1.2	27.40 ± 1.24	0.073
TICV (mm^3^)	1415.40 ± 103.40	1395.26 ± 162.12	0.678
Disease duration (years)	—	2.59 ± 2.08	—
UPDRS (part III)	—	15.63 ± 5.81	—
H&Y stage	—	1.72 ± 0.63	—
Dominantly affected side (right : left)	—	12 : 4	—
Right handed	16	16	—

Data are the mean ± standard deviation in age, MMSE scores, and TICV. The *p* values for comparison in age, MMSE scores, and TICV were obtained by independent *t*-tests. The *p* values for distribution of sex were obtained by chi-square tests. HC, healthy control group; PD, Parkinson's disease group; MMSE, mini-mental status examination; TICV, total intracranial cavity volume.

**Table 2:** The differences of the total and subfield hippocampal volumes between the groups determined by the diagnosis and by the hemisphere.

Subfield	Volume (mm^3^)				Diagnosis		Hemisphere		Diagnosis X hemisphere	
	HC		PD							
	Right	Left	Right	Left	*F*	*p*	*F*	*p*	*F*	*p*
CA1	316.88 ± 35.73	323.80 ± 44.40	316.59 ± 32.91	305.31 ± 33.55	0.680	0.417	0.374	0.546	1.732	0.199
CA2-3	905.50 ± 109.62	907.39 ± 153.15	922.81 ± 103.26	851.60 ± 87.20	0.308	0.584	1.946	0.174	7.586	**0.010**
CA4-DG	509.05 ± 53.49	514.72 ± 87.24	520.51 ± 58.62	480.69 ± 50.33	0.419	0.523	0.042	0.839	7.403	**0.011** ^*∗*^
Presubiculum	429.68 ± 68.80	441.29 ± 80.29	402.54 ± 73.23	407.71 ± 70.40	1.900	0.179	0.143	0.708	0.122	0.730
Subiculum	594.36 ± 61.26	603.78 ± 68.22	579.25 ± 84.45	553.55 ± 75.52	2.398	0.133	1.274	0.269	2.998	0.095
Fimbria	46.48 ± 19.80	44.86 ± 21.47	38.21 ± 19.45	38.95 ± 21.18	0.880	0.356	0.001	0.971	0.107	0.747
Fissure	46.38 ± 22.34	39.14 ± 21.59	46.18 ± 15.15	32.61 ± 12.88	0.200	0.658	1.879	0.182	0.878	0.357
Total	2801.93 ± 280.20	2835.81 ± 388.63	2779.93 ± 323.33	2637.83 ± 277.87	1.379	0.250	0.084	0.774	5.197	**0.031**

Data are the mean ± standard deviation (mm^3^). Repeated-measures analysis of covariance (ANCOVA) controlling for age, sex, and total intracranial cavity volume was performed. “Diagnosis X hemisphere” means the interaction effect of diagnosis by hemisphere. FDR was applied in the comparisons of the total and subfield volumes (8 comparisons), *q* < 0.05. ^*∗*^Regions that remained significant after FDR correction are marked with an asterisk. HC, healthy control group; PD, Parkinson's disease, nondementia group; CA, cornu ammonis; DG, dentate gyrus; FDR, false discovery rate by Benjamini and Hochberg.

**Table 3:** Post hoc analysis in the differences of the total and subfield hippocampal volumes between the hemispheres in the diagnosis groups.

Subfield	Volume (mm^3^)							
	HC				PD			
	Right	Left	*F*	*p*	Right	Left	*F*	*p*
CA2-3	905.50 ± 109.62	907.39 ± 153.15	0.005	0.946	922.81 ± 103.26	851.60 ± 87.20	19.911	**<0.001** ^*∗*^
CA4-DG	509.05 ± 53.49	514.72 ± 87.24	0.127	0.726	520.51 ± 58.62	480.69 ± 50.33	20.273	**<0.001** ^*∗*^
Total	2801.93 ± 280.20	2835.81 ± 388.63	0.234	0.635	2779.93 ± 323.33	2637.83 ± 277.87	10.573	**0.005** ^*∗*^

Data are the mean ± standard deviation (mm^3^). Repeated-measures analysis of variance tests were performed. HC, healthy control group; PD, Parkinson's disease, nondementia group; CA, cornu ammonis; DG, dentate gyrus. ^*∗*^A significant statistical difference with *p* < 0.05.

## Data Availability

The data that support the findings of this study are available on request from the corresponding author. The data are not publicly available due to information that could compromise the privacy of research participants.
